# Smart Graphite–Cement Composites with Low Percolation Threshold

**DOI:** 10.3390/ma15082770

**Published:** 2022-04-09

**Authors:** Maksymilian Frąc, Paulina Szołdra, Waldemar Pichór

**Affiliations:** Department of Building Materials Technology, Faculty of Materials Science and Ceramics, AGH University of Science and Technology, Al. Mickiewicza 30, 30-059 Krakow, Poland; szoldra@agh.edu.pl (P.S.); pichor@agh.edu.pl (W.P.)

**Keywords:** cement composites, electrical properties, dispersion, piezoresistive properties, graphite, self-sensing, structural health monitoring

## Abstract

The objective of this work was to obtain cement composites with low percolation thresholds, which would reduce the cost of graphite and maintain good mechanical properties. For this purpose, exfoliated graphite was used as a conductive additive, which was obtained by exfoliating the expanded graphite via ultrasonic irradiation in a water bath with surfactant. To obtain evenly distributed graphite particles, the exfoliated graphite was incorporated with the remaining surfactant into the matrix. This study is limited to investigating the influence of exfoliated graphite on the electrical and mechanical properties of cement mortars. The electrical conductivity of the composites was investigated to determine the percolation threshold. The flexural and compressive strength was tested to assess the mechanical properties. In terms of the practical applications of these composites, the piezoresistive, temperature–resistivity, and thermoelectric properties were studied. The results showed that the incorporation of exfoliated graphite with surfactant is an effective way to obtain a composite with a percolation threshold as low as 0.96% (total volume of the composite). In addition, the mechanical properties of the composites are satisfactory for practical application. These composites also have good properties in terms of practical applications. As a result, the exfoliated graphite used can significantly facilitate the practical use of smart composites.

## 1. Introduction

In recent years, smart materials have been used in practically every field of science and technology. Cement composites in the form of concrete and mortar could also be used as smart materials. However, traditional cement composites are materials whose electrical conductivity is small, and their electrical properties are influenced by factors such as moisture, temperature, and hydration time [1,2,3]. For example, the resistivity of the dried cement matrix is in the order of 10^8^ Ω·cm, while the resistivity of the matrix saturated with water is approximately 10^3^ Ω·cm [1]. In addition, changes in the electrical parameters of these cement composites under the influence of various external factors are small. For these reasons, the use of traditional cement composites as smart materials is not possible.

On the other hand, such possibilities are provided by the introduction of electrically conductive materials such as carbon fibers [4,5,6,7,8,9,10], carbon nanotubes [11,12,13,14], graphite-based particles [15,16,17,18,19,20,21,22,23,24,25,26], steel fibers [27,28,29,30], shungite [31], and metal particles [32,33] into the cement matrix. Cement composites with these conductive additives can change resistivity under the influence of mechanical stress (piezoresistive effect). Therefore, these composites can be used as stress monitoring sensors (for structural vibration control, traffic monitoring, and weighing) and for damage detection [7,10,12,13,14,20,33]. These composites can be applied as temperature sensors (thermal control, hazard mitigation, structural performance control) using the Seebeck effect [9,17,18,23], or the phenomenon of resistivity changes with temperature [22,24]. These cement composites can also be utilized as heating elements (for surface de-icing, underfloor heating) or to shield electromagnetic radiation [34,35,36,37].

In terms of the practical use of these composites, the main challenge is to obtain good electrical properties without deterioration of the mechanical strength and durability of the composites. Good electrical properties are obtained when the percolation threshold of the conductive additive in the composite is exceeded, i.e., when the conductive additive particles form the conductive network throughout the matrix [9,20]. For materials such as graphite powder [24], nickel particles [32], and shungite [31], a large amount of these materials must be introduced to exceed the percolation threshold. As a result, it leads to a reduction in mechanical strength and an increase in production costs. Therefore, the percolation threshold must be exceeded with a low additive content. The introduction of carbon fibers, nanotubes, and graphite nanoparticles allows the percolation threshold to be reached with a low addition of 0.5–2 vol% (in relation to the total volume of the composite) [5,13,19]. However, apart from the high price, the main disadvantage of these materials is their tendency to agglomeration, which makes their practical application more difficult. Their tendency to agglomerate leads to the formation of aggregated particles in the matrix. Consequently, a higher additive content is required to achieve desirable electrical properties, and, thus, the mechanical properties of composites are reduced. Therefore, the uniform dispersion of conductive additives is a key issue in the case of cement composites [37,38,39]. There are several methods for improving the dispersion of conductive additives in the cement matrix. The most popular method is the introduction of conductive additives with surfactants [37,38,40,41]. Ultrasonication and mechanical stirring are generally used in combination with the addition of surfactants to obtain optimal dispersion [37,38,39,42]. Another method is to use admixtures such as silica fume, latex, acrylic, or silanes [30,43,44,45,46]. The dispersion of conductive additives is also improved by increasing the hydrophilicity of particles by surface modification [8,47,48]. However, all of these methods impede the preparation process and increase costs.

One of the promising materials that can be used as a conductive additive is expanded graphite [20,21,22,23]. It is a relatively inexpensive material that can be easily obtained by rapidly heating intercalated graphite. The introduction of expanded graphite into the cement matrix allows for the obtaining of a percolation threshold with a relatively low addition of 5% (in relation to cement). However, the mechanical strength of these composites is reduced because the expanded graphite grains are long and porous [20,21]. The method of reducing the porosity of expanded graphite is its exfoliation to smaller nonporous particles. This is possible because the bonds between the carbon layers in the expanded graphite are weak, allowing them to be separated easily. There are many methods of exfoliation of expanded graphite [21,49,50,51,52,53,54,55]. The most effective method is the exfoliation of expanded graphite by ultrasonic irradiation. This inexpensive method involves immersing grains of expanded graphite in a liquid such as acetone, alcohols, and benzene and then subjecting them to high-frequency waves [50,51,52,53,54,55]. According to [50,51,53], this method allows the acquisition of graphite particles with thicknesses in the order of nanometers and an aspect ratio in the range of 400–7000. As a result, the use of such particles in cement composites may allow for the creation of conductive networks with their small addition.

The main objective of this work was to obtain cement composites with a low percolation threshold, which would reduce the cost of graphite and maintain good mechanical properties. For this purpose, exfoliated graphite was used as a conductive additive, which was obtained by an inexpensive and easy method of exfoliation of expanded graphite. This method included exfoliation of expanded graphite by ultrasonic irradiation in a water bath with the addition of surfactant, which was used to reduce surface tension. The exfoliated graphite obtained could be an alternative to the expensive commercially available graphite nanoparticles [15,16,17,18,19,56]. Until now, no research has been published on the use of exfoliated graphite obtained by this method in cement composites. An additional novelty of this work is the introduction of the remaining surfactant after exfoliation in combination with the exfoliated graphite into the cement matrix to obtain evenly distributed graphite particles. This approach eliminates the additional process for optimal dispersion during the cement composite preparation step. The authors of [57] use a similar approach, but the graphite nanoparticles (exfoliated graphene oxide) were obtained from natural graphite flakes using a chemical method. Furthermore, an additional process was used during the preparation of the composites for optimal dispersion.

To investigate the effect of the introduction of exfoliated graphite on the electrical properties of cement composites, the following research was carried out. The electrical conductivity of the composites was investigated to determine the percolation threshold. The flexural and compressive strengths were tested to assess the mechanical properties. In terms of practical applications of these composites, the piezoresistive, temperature–resistivity, and thermoelectric properties were studied. Furthermore, the obtained size of the graphite particles and their dispersion in the cement matrix were investigated.

This research in this work is limited to electrical and mechanical properties. Therefore, further research is required, mainly on the rheology of the fresh mix and the microstructure of the composite, with particular emphasis on the interfacial transition zones between graphite and the cement matrix.

## 2. Experimental

### 2.1. Materials

Expanded graphite (Sinograf) with a grain size in the range of 2–4 mm and a density of 4.3 kg/m^3^ was used for the exfoliation process. Portland cement CEM I 42.5 and quartz sand with grain sizes of less than 0.5 mm were utilized. A commercial mixture of surfactants (anionic, nonionic, and amphoteric compounds) was used. The surface tension of the surfactant mixture was approximately 51 mN/m, measured using the stalagmometric method at a constant temperature of 20 °C.

### 2.2. Preparation of Exfoliated Graphite

The expanded graphite was exfoliated by ultrasonic irradiation in a water bath with the addition of a surfactant, which was intended to facilitate the exfoliation of graphite by reducing the surface tension of the water. The exfoliation process was carried out using ultrasonic waves with a frequency of 40 kHz and a power of 160 W. The irradiation time was 1 h. The mass ratio of water-graphite was 600. The surfactant was added in an amount to obtain the desired amount in the composite (1 wt% in relation to cement). The suspension of exfoliated graphite obtained with surfactant was heated to evaporate water to the amount resulting from the adopted water-cement ratio (0.4) in the cement composite.

### 2.3. Preparation of Cement Composites with Exfoliated Graphite

Cement mortars with different amounts of exfoliated graphite were prepared to study their electrical and mechanical properties. Mortars were prepared according to the PN-EN 196-1:2006 standard. Exfoliated graphite was introduced into the matrix in a ratio of 1 to 4 by cement mass to study the effect of the amount of addition on the properties of the composites. For comparison of the degree of dispersion, mortars with exfoliated graphite without surfactant were made. In addition, a reference mortar without conductive additives was prepared. The cement/water and sand/cement ratios were 0.4 and 0.75, respectively (Table 1). Samples with dimensions of 15 mm × 15 mm × 75 mm were cast for all tests (except spectroscopy impedance measurements). For the measurement of electrical properties, copper sheets were attached to the surface of both ends of the samples, which ensured a connection to the measuring equipment. Silver paste was applied to the ends of the samples to reduce the influence of contact resistance. The cement mortars were stored in water, and the experiments were carried out after 28 days of curing. Before electrical properties tests, the samples were dried to a constant mass (temperature of 60 °C). For all studies, three samples for each composite were measured.

### 2.4. Experimental Method and Equipment

#### 2.4.1. Characterization of Exfoliated Graphite and Its Dispersion in Matrix

The particle size distribution of the exfoliated graphite was measured using the laser diffraction method (Malvern Mastersize 2000, Malvern, UK). Furthermore, the particle sizes were also examined by SEM (Nova NanoSEM 200, FEI, Hillsboro, OR, USA) observation of exfoliated graphite in mortars. The dispersion of exfoliated graphite in the cement matrix was observed using the VHX-7000 digital microscope (Keyence, Japan).

#### 2.4.2. Mechanical Properties of Composites

The compressive and flexural strength was determined to investigate the effect of the incorporation of exfoliated graphite on the mechanical properties of the composites. The tests were conducted according to the PN-EN 196-1: 2006 standard. The compressive and flexural strength was tested at a deformation rate of 2.4 kN/s and 0.05 kN/s, respectively.

#### 2.4.3. Electrical Conductivity of Composites

Resistivity measurements were performed to study the effect of the amount of exfoliated graphite on the electrical conductivity of composites. Resistance was measured with an LCR meter (Agilent U1733C) using a two-probe AC method at a frequency of 10 kHz. From the data obtained, the resistivity was calculated using the formula [9]:

(1) $$\rho =\frac{R\cdot A}{l},\Omega \cdot \mathrm{cm},$$

where *R* is the resistance, *A* is the cross-sectional area, and *l* is the length.

Impedance spectroscopy (IS) measurements were applied to determine the percolation threshold in the composites. For this purpose, the Solartron SI1255 analyzer (with a CDI interface) was used in the range of 1 × 10^−1^–10^5^ Hz. For these measurements, samples were prepared with dimensions of 15 mm × 15 mm × 15 mm. To reduce the contact resistance, a silver layer was applied to the contact surface between the measuring electrode and the sample. Data analysis was performed with WinFIT software (Novocontol). Four samples of each compound were measured. The IS results were presented in the representation of Z″ = f (Z′) (Nyquist plots), where Z′ and Z″ are the real and imaginary parts of the impedance, respectively.

#### 2.4.4. Piezoresistive Properties of Composites

**Figure 1:**
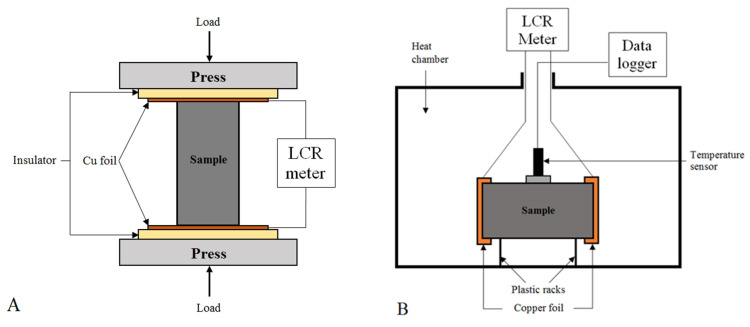
Experimental setup for (**A**) piezoresistive and (**B**) resistivity–temperature properties study.

The piezoresistive properties were determined in terms of the possibility of using composites with exfoliated graphite for stress monitoring. For this purpose, the changes in the resistance of the samples under cyclic compression loading were measured. Figure 1A presents the experimental setup for this study. Resistance was measured using an LCR meter (Agilent U1733C) using a two-probe AC method at a frequency of 10 kHz. The samples were subjected to five loading cycles at a rate of 4 mm/min. The composites were loaded to 50% of their compression strength. For each cycle, the minimum and maximum loads were kept for 10 s. The load (*F*) was automatically recorded, and then the stress (*K*) was calculated from the formula [31]:

(2) $$K=\frac{F}{A},\mathrm{MPa},$$

where *A* is the cross-sectional area.

The resistance value was converted to resistivity using Equation (1).

Changes in resistivity were presented as fractional changes in resistivity (*FCR*), which was calculated from the following equation [31]:

(3) $$FCR=\frac{\rho -\rho_{0}}{\rho_{0}}\cdot 100\%,$$

where *ρ* is the resistivity of the composite during loading, and *ρ*_0_ is the initial resistivity before test.

The strain sensitivity (gauge factor) and the stress sensitivity were determined from the data obtained. Since the dimensional changes of the cement composite during compression are negligible, the measured resistance remains essentially proportional to the resistivity [6]. As a result, the gauge factor (GF) [6] and the stress sensitivity (*F*) [31] were calculated from the equations:

(4) $$\mathrm{GF}=\frac{\left(\rho -\rho_{0}\right)/\rho_{0}}{\varepsilon }$$

(5) $$F=\frac{FCR}{\sigma },\%/\mathrm{MPa},$$

where *FCR* is the fractional change in resistivity, *ε* is the strain, a *σ* is the stress.

#### 2.4.5. Temperature–Resistivity Properties of Composites

Temperature–resistivity properties were characterized in terms of the possibility of using the composites with exfoliated graphite as a temperature sensor. Temperature–resistivity property measurements were made according to the method used in [22]. Figure 1B shows the experimental setup used for this study. The resistance of the samples was measured during heating and cooling in the range of 20–60 °C. An LCR meter (Agilent U1733C) using the two-probe AC method at the frequency of 10 kHz was used to take resistance measurements. The resistance values were converted to resistivity using Equation (3). The changes in resistivity were presented as fractional changes in resistivity (FCR), which were calculated from Equation (3), where *ρ* is the resistivity of the composite during the test, and *ρ_o_* is the resistivity at 20 °C.

The resistivity temperature coefficient of resistivity (*α*) was also calculated according to the formula [22]:

(6) $$\alpha =\frac{\left(\rho -\rho_{o}\right)}{\rho_{o}\cdot \Delta T},\;1/K,$$

where Δ*T* is the temperature difference relative to 20 °C.

#### 2.4.6. Thermoelectric Properties of Composites

**Figure 2:**
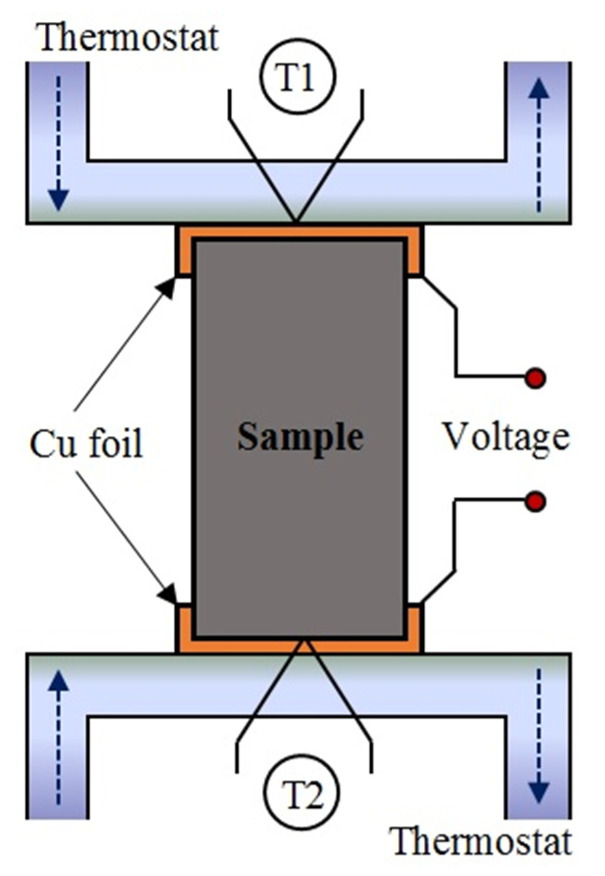
Experimental setup for the determination of thermoelectric properties.

Thermoelectric properties were also investigated to examine the possibility of using composites with exfoliated graphite as a temperature sensor. Thermoelectric properties were studied according to the method used in [22,31]. The experimental setup is shown in Figure 2. The temperature gradient between both ends of the samples was in the range of 0 to 60 °C relative to the temperature of 20 °C. The thermoelectric voltage generated by the samples was measured during heating and cooling. Voltage measurements were performed with an ESCORT 3145A multimeter. The Seebeck coefficient (in relation to copper) was estimated using the following formula [22]:

(7) $$S=\frac{\Delta V}{\Delta T},\frac{V}{K},$$

where Δ*V* is the measured voltage, and Δ*T* is the temperature gradient between the ends of the sample.

## 3. Results and Discussion

### 3.1. Characterization of the Particle Size of Exfoliated Graphite

**Figure 3:**
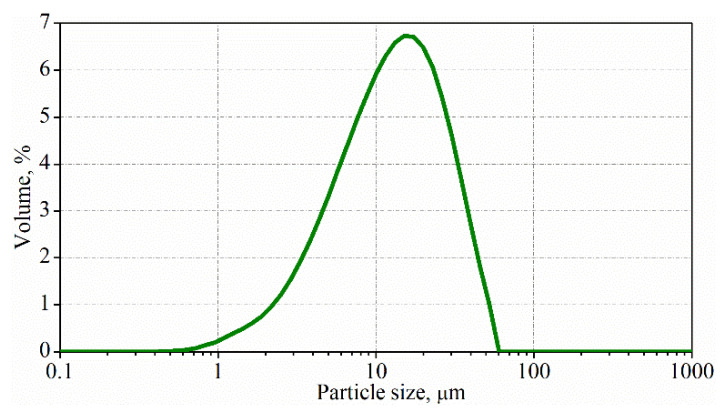
Particle size distribution after exfoliation of expanded graphite.

The measurement of the particle size distribution showed that the particle size of the obtained graphite is less than 40 µm, and the modal value is about 15 µm (Figure 3). Additionally, particles smaller than 1 µm are achieved. The size of the expanded graphite before exfoliation was in the range of 2 to 4 mm.

**Figure 4:**
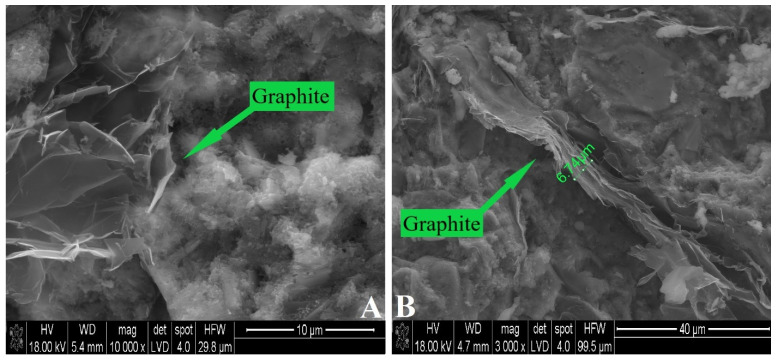
SEM images of cement composites with graphite content: (**A**) 2 wt% and (**B**) 3 wt%.

The SEM observations confirmed that there are particles with dimensions in the order of micrometers or even less in the cement matrix (Figure 4). This observation also revealed that the obtained particles are in the form of sheets of larger diameter and smaller thickness. As a result, the particles have a high aspect ratio, which may facilitate the formation of conductive networks in the matrix.

The results of particle size characterization revealed that the method of exfoliation of expanded graphite by ultrasonic irradiation into the water with a surfactant is an effective method for breaking expanded graphite into micrometer particles with a high aspect ratio. The particle sizes obtained are similar to those found in works in which other liquids were used during the exfoliation of expanded graphite [50,51,52,53].

### 3.2. Dispersion of Exfoliated Graphite in Cement Matrix

**Figure 5:**
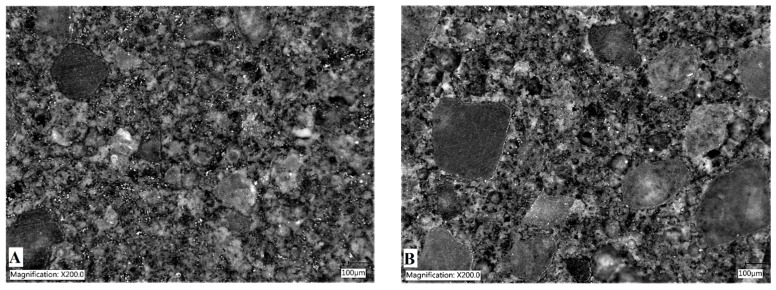
Microscopy images of composites with graphite content: (**A**) 2 wt% and (**B**) 1 wt%; graphite particle—black color, cement matrix and sand—lighter color.

Figure 5 shows microscope images of the composites with exfoliated graphite. As can be seen in the images, the exfoliated graphite is evenly distributed in the cement matrix and no graphite agglomerates are visible. Furthermore, graphite particle networks are noticeable for composites with a graphite content greater than 2 wt% (Figure 5A). Otherwise, in the case of the composite with a content of 1 wt%, there are no visible graphite networks, but there are evenly distributed and separated graphite particles (Figure 5B).

The results show that the use of the surfactant remaining from the exfoliation process is an effective method to obtain well-dispersed graphite particles. This approach eliminates the need to introduce additional processes during the preparation of composites. As a consequence, the use of exfoliated graphite with a surfactant can significantly facilitate the practical use of smart composites.

### 3.3. Mechanical Properties of Composites

**Figure 6:**
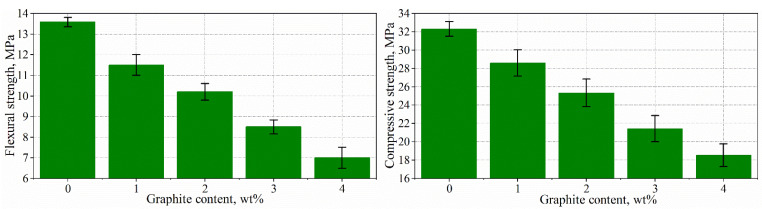
Flexural and compressive strengths of composites with exfoliated graphite.

The results of compressive and flexural strength measurements are shown in Figure 6. As can be seen in Figure 6, the introduction of exfoliated graphite into the matrix leads to the deterioration of the mechanical strength of cement composites. The flexural and compressive strength values of the composites with the addition of 1 wt% are less than approximately 15 and 12% of the reference composites without the addition of exfoliated graphite. The higher the graphite content, the lower the strength values. For the composites with an addition of graphite 4 wt%, the flexural and compressive strength values are reduced by approximately 48 and 43% compared to the reference sample.

The decrease in mechanical properties is likely related to the increase in porosity due to the introduction of air by the surfactant application and the weak bond between the exfoliated graphite and the matrix. However, the values of the mechanical strength are satisfactory in terms of practical applications. Furthermore, the mechanical strength values are significantly higher than those of the cement composites with the addition of expanded graphite. For example, the compressive strength value of composites with the addition of 4 wt% of exfoliated graphite is 18.5 MPa, while the compression strength of composites (w/c ratio = 0.5, s/c ratio = 0.75) with 4 wt% expanded graphite is approximately 10 MPa [21].

### 3.4. Electric Conductivity of Composites

**Figure 7:**
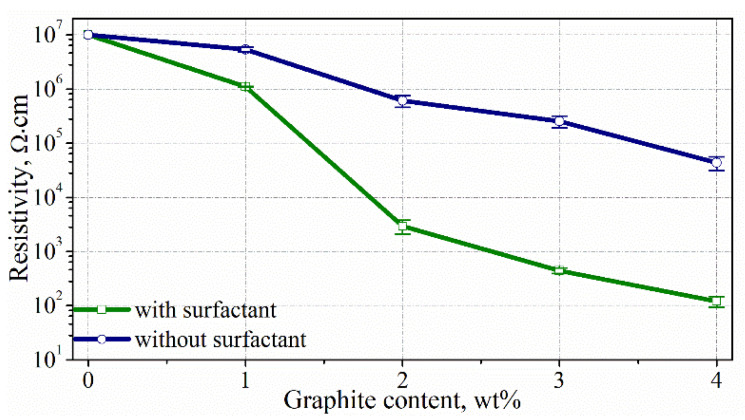
Relationship between resistivity and graphite content for composites with (**green line**) and without (**blue line**) surfactant.

Figure 7 reveals a relationship between the resistivity of the composites and graphite content. As can be observed in the figure, the introduction of exfoliated graphite reduces the resistivity of the composite. The greater the addition of graphite, the lower the resistivity of the composite. For composites with the addition of 1 wt% of graphite, the resistivity reduced slightly compared with the composite without graphite. Instead, there is a significant drop in resistivity for the composite with a content of 2 wt%. The introduction of a higher graphite content causes a gradual reduction in resistivity, but these changes are relatively small.

Figure 7 also presents the measured resistivity for surfactant-free composites. The resistivity values of the surfactant-free composites are significantly higher than those of the composites with surfactant. For the content of 4 wt% graphite, the resistivity is about 0.1 and 44.0 kΩ·cm for the composites with and without surfactant, respectively. These results confirm the good dispersion of exfoliated graphite in the matrix when a surfactant is used. This difference is related to the fact that the better dispersion associated with the use of surfactant causes the formation of conductive networks of graphite at significantly lower graphite addition.

Based on the results of the resistivity measurements, it can be assumed that the percolation threshold occurs in the range of 2–4 wt% of graphite for the composites with a surfactant. However, it is difficult to determine the exact graphite content at which the percolation threshold is exceeded. According to [20], impedance spectroscopy (IS) can be used to precisely determine the percolation threshold in cement composites. On the basis of this method, the percolation threshold occurs when the reactance of the composite changes from captative to inductive. Therefore, IS was used in this study to determine the percolation threshold.

**Figure 8:**
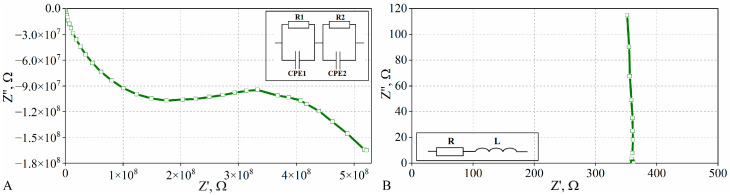
Nyquist plots and adopted equivalent circuits for composites with graphite content of (**A**) 1 wt% and (**B**) 2 wt%.

Impedance spectroscopy measurements revealed that two types of Nyquist plots are observed, which indicate a different character of reactance. In the impedance spectra, for the composite with a 1 wt% graphite content, a high-frequency semicircle followed by an arc at lower frequencies (Figure 8A) is visible. This indicates the captative character of the reactance of the sample [20]. On the basis of the Nyquist plot, the equivalent circuit was fitted (Figure 8A). The first loop includes a resistor (R1) and a constant phase element (CPE1) connected in parallel, which is responsible for the resistance inside the graphite particles and dispersion of the intergranular boundaries (corresponding to a high-frequency semicircle). The second loop (composed of R2 and CPE2) corresponds to the electrode processes on both ends of the sample. These results show that there are gaps between adjacent graphite particles, and, as a consequence, the percolation threshold is not reached.

In the case of the composites with a graphite content above 2 wt%, the impedance spectra look quite different than those of composites with a lower amount of graphite. The observed curve is almost vertical (Figure 8B), indicating changes in the character of the reactance to the inductance. On the basis of the impedance spectra, the second equivalent circuit consisting of a resistor (R) and a series-connected inductor (L) was fitted (Figure 8B). Such an equivalent model is typical for situations where a continuous network of graphite particles is present throughout the matrix. This means that the percolation threshold is exceeded for composites with the addition of 2 wt%.

The results of the electrical conductivity measurements indicate that the method of exfoliation of expanded graphite is effective for obtaining graphite particles, which allow for the achievement of a percolation threshold of the cement composite at additions as low as 0.96 vol% of the total volume of the composite (2 wt% cement). This amount is significantly lower than those measured for composites with expanded graphite incorporated directly into the cement matrix (5% by weight of cement) [20]. Furthermore, the content required to obtain the percolation threshold is similar to or even lower than that of cement composites with expensive, commercially available graphene nanoplatelets. In [15], the percolation threshold is reached for cement composites with 2.4 vol% of graphene nanoplatelets (to the total volume of the composite). A similar value is obtained in [19], the percolation threshold is achieved at a content of 2 vol% of the total volume of the composite. On the contrary, the authors in [16] exceeded the percolation threshold with a content of 1.2 wt% (in relation to cement). Furthermore, as can be seen in Table 2, the percolation threshold for composites with carbon fibers [6,9] and nanotubes [13,34] is reached at lower contents, while it was reached at significantly higher contents for composites with the addition of shungite [31] and carbon black [25].

### 3.5. Piezoresistive Properties of Composites

**Figure 9:**
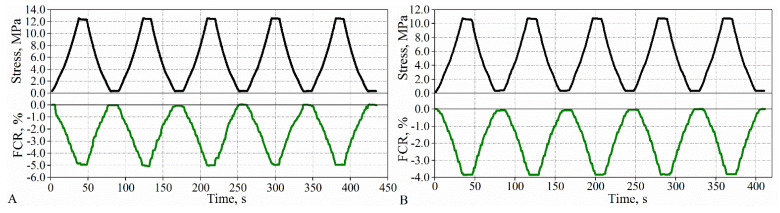
Relationship between FCR and stress under cyclic loading for composites with graphite content: (**A**) 2 wt% and (**B**) 3 wt%.

Measurements of piezoresistive properties showed that cement composites with a graphite content greater than 2 wt% exhibit measurable resistivity changes with variations in stress. The resistivity of the composites decreases linearly upon loading, while the resistivity increases linearly upon unloading (Figure 9). The reduction in resistivity of the composites is related to the approach of the adjacent graphite particles toward each other during loading. On the other hand, upon unloading, the distance between the particles increases, and, thus, the resistivity increases [21,31]. Furthermore, for these composites, changes in resistivity in subsequent cycles correspond to variations in stress. Figure 9 shows typical graphs for composites with graphite content above the percolation threshold. As can be seen, the resistivity values are nearly the same in subsequent cycles, and the resistivity at the end of each cycle is equal to the initial resistivity before loading.

**Figure 10:**
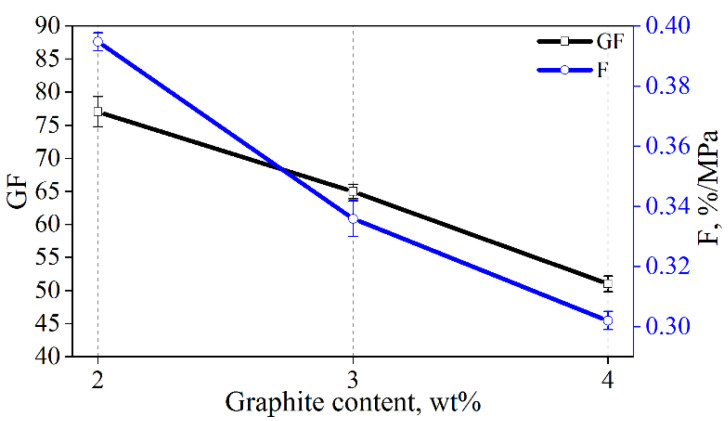
Gauge factor (GF) and stress sensitivity (F) of composites with exfoliated graphite.

The calculated sensitivity values of the composites with exfoliated graphite are presented in Figure 10. It can be seen that the values for both the gauge factor and the stress sensitivity decrease with increasing graphite content. The GF values are 77, 65, and 51 for composites with 2, 3, and 4 wt% of graphite, respectively. The stress sensitivity decreases as follows: 0.39, 0.34, and 0.30 with an increasing amount of graphite. This phenomenon of a reduction in sensitivity with an increase in the content of conductive additives is consistent with the results of cement composites with other conductive additives [25,30].

The sensitivity values of the composites with exfoliated graphite are high compared to composites with conductive materials such as expanded graphite [21], carbon fiber [6], carbon black [26], and shungite [31] (Table 3). On the contrary, the sensitivity of the composites with exfoliated graphite is lower than that of composites with materials such as carbon nanotubes [14], nanographite [19], and steel fibers [28].

The results of the study of the piezoresistive properties revealed that the use of exfoliated graphite allows the application of cement composites as a sensor for stress monitoring. Good piezoresistive properties are obtained for a content as low as 0.96 vol% of the total volume of composites (2 wt% cement). These composites possess relatively high sensitivity and present linear changes in resistivity with variation in stress. Moreover, these composites exhibit good synchronization of changes in resistivity with variations in stress in subsequent measurements.

### 3.6. Temperature–Resistivity Properties of Composites

**Figure 11:**
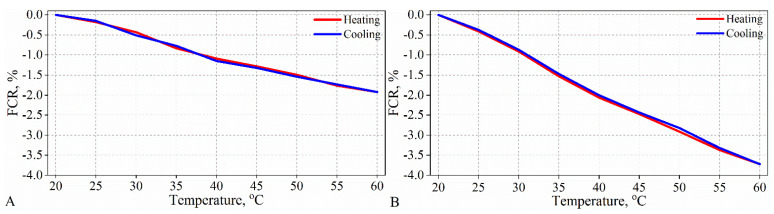
Fractional change in resistivity with temperature during heating and cooling for composites with graphite content: (**A**) 2 wt% and (**B**) 3 wt%.

The results of the study of the temperature–resistivity properties reveal that cement composites with exfoliated graphite show a negative temperature coefficient of resistivity, that is, the resistivity of the composite decreases with increasing temperature (Figure 11). Composites with the addition of exfoliated graphite above 2 wt% exhibit linear changes in resistivity with temperature during both heating and cooling, and the heating–cooling curves almost completely coincide, as exemplified in Figure 11.

**Figure 12:**
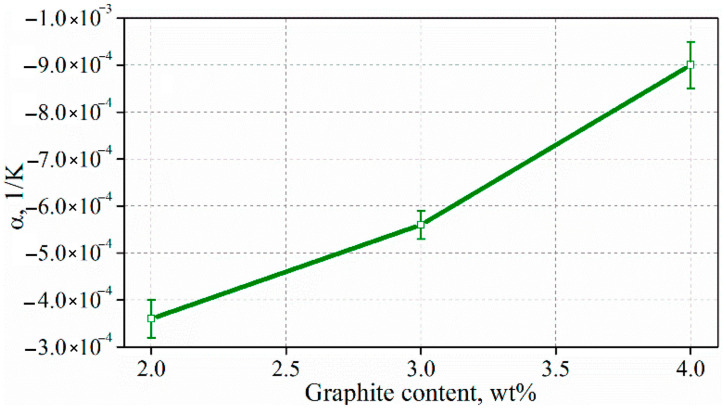
Temperature coefficient of resistivity for cement composites with exfoliated graphite.

The calculated values of the temperature coefficient of resistivity showed that the higher the graphite content in the composite, the higher value of the temperature coefficient of resistivity (Figure 12). The highest value of the temperature coefficient is −9 × 10^−4^ 1/K. These values are relatively high and comparable to the values obtained for cement composites with expanded graphite (−4–−12 × 10^−4^ 1/K) [22].

The results of this study reveal that cement composites with exfoliated graphite possess good temperature–resistivity properties. Composites have a relatively high value of the temperature coefficient of resistivity and very high stability and repeatability of changes in resistivity as a function of temperature. For these reasons, composites with as low graphite addition as 0.96 vol% (the total volume of composite) can be used as temperature sensors, which can be used to control shutters that obscure sunlight or to control heating systems.

### 3.7. Thermoelectric Properties of Composites

**Figure 13:**
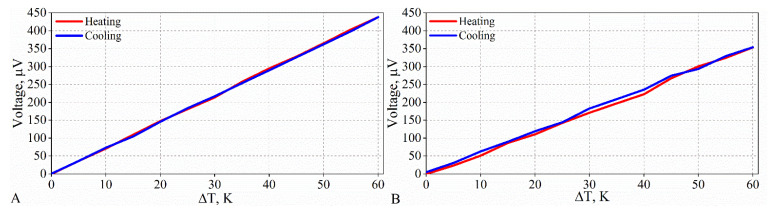
Changes in thermoelectric voltage with temperature gradient during heating and cooling of composites with content of (**A**) 3 wt% and (**B**) 2 wt%.

The thermoelectric property measurements showed that a measurable Seebeck effect occurs at a graphite content greater than 2 wt%. The thermoelectric voltage generated by these composites changes linearly with the temperature gradient during both heating and cooling (Figure 13). However, the measurability of the voltage changes with the temperature gradient, depending on the graphite content. The heating–cooling curves almost perfectly overlap for composites with a graphite content greater than 3 wt% (Figure 13A). However, for the composite with a 2% graphite content, the heating and cooling curves do not coincide well (Figure 13B).

**Figure 14:**
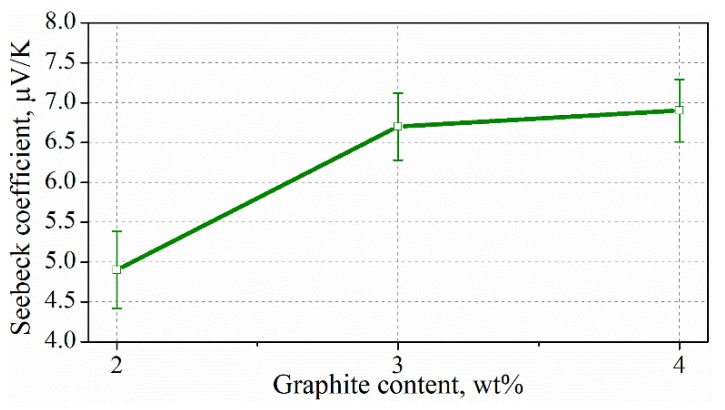
Seebeck coefficient of cement composites with exfoliated graphite.

Figure 14 shows the calculated Seebeck coefficient of cement composites with exfoliated graphite. As can be seen, the Seebeck coefficients of the composites with 3 and 4 wt% of addition are similar, and the values are 6.7 and 6.9 μV/K, respectively. However, for the composite with 2 wt% of graphite content, the value of the Seebeck coefficient is lower (4.9 μV/K). On the basis of the results obtained, it can be concluded that the higher the Seebeck coefficient, the more reproducible changes in the thermoelectric voltage with a temperature gradient.

Table 4 shows the comparison of the Seebeck coefficients for cement composites with different conductive additives. As can be seen, the Seebeck coefficient of exfoliated graphite is relatively small compared to the composites with other conductive additives, such as carbon nanotubes (57.9 μV/K) [11], steel fibers (59 μV/K) [29], graphite powder (18 μV/K) [24], and expanded graphite obtained at 500 °C (13–15 μV/K) [22]. On the other hand, the obtained values of the Seebeck coefficient are higher than the values for cement composites with expanded graphite obtained at 1000 °C (3.0 μV/K) [22] and shungite (4.1 μV/K) [31].

The results of the thermoelectric properties study reveal that cement composites with a graphite content greater than 1.43 vol% (total volume of the composite) can be used as temperature sensors for some smart applications. This is due to the fact that, despite the low Seebeck coefficient, the voltage changes that occur along the temperature gradient are linear for these composites. Additionally, voltage changes are reproducible for heating and cooling. For example, these composites can be used to monitor the temperature of building partitions, because large values of thermoelectric voltage are not required for this purpose.

## 4. Conclusions

Based on the results obtained, the following conclusions can be drawn:

- Exfoliation of expanded graphite in water with surfactant by ultrasonic irradiation is an effective method to obtain smaller graphite particles with a size of less than 40 µm.
- The addition of the surfactant remaining after exfoliation to the exfoliated graphite during the preparation of the composite allows for a good dispersion of the exfoliated graphite into the composites.
- The addition of exfoliated graphite to the cement matrix allows the percolation threshold with a low graphite addition of 0.96 vol% of the total volume of composite (2 wt% cement) to be reached.
- The incorporation of exfoliated graphite reduces the mechanical strength of the composites. However, the mechanical properties are satisfactory for practical application. The flexural and compressive strength values of the composites with a graphite addition of 0.96 vol% (above the percolation threshold) are 10.2 and 25.3 MPa, respectively.
- The cement composites with exfoliated graphite have good synchronization and repeatability of resistivity changes with variations in stress and possess relatively high values of sensitivity.
- The cement composites with the addition of exfoliated graphite above the percolation threshold exhibit a relatively high temperature coefficient of resistivity (−9 × 10^4^ 1/K), and the changes in resistivity with temperature variation are linear and repeatable.
- The cement composites with the addition of exfoliated graphite greater than 1.43 vol% (of the total volume of composite) show linear and repeatable changes in thermoelectric voltage during heating and cooling, and the highest Seebeck coefficient is 6.9 μV/K.

In general, these results indicate that exfoliated graphite, which can be obtained via an easy and inexpensive method, is a promising conductive additive for cement composites. Furthermore, the introduction of the surfactant remaining after exfoliation, along with the exfoliated graphite, simplifies the preparation of the composition. As a result, this can significantly facilitate the practical use of smart composites. Due to their good piezoresistive properties, such composites can be used for stress monitoring, which can be applied to assess the load condition of a building structure or to weigh vehicles moving through concrete pavements. Good temperature–resistance properties allow the compounds to be utilized as temperature sensors, which can be used to control shutters obscuring sunlight or to control heating systems. The thermoelectric properties of these composites allow them to be applied to monitor the temperature of building partitions.

However, further studies are required to fully characterize composites with exfoliated graphite, mainly looking into the rheology of the fresh mix and the microstructure of the composite, with particular emphasis on the interfacial transition zones between graphite and the cement matrix.

## Figures and Tables

**Table 1:** Proportions of prepared cement composites.

Content of Exfoliated Graphite, by Weight of Cement, %	Content of Exfoliated Graphite in Relation to the Total of the Composite, %	w/c Ratio	s/c Ratio
0	-	0.40	0.75
1	0.49		
2	0.96		
3	1.43		
4	1.90		

**Table 2:** Percolation thresholds for cement composites with different conductive additives.

Conductive Additive		Percolation Threshold	Reference
Exfoliated graphite		2 wt% of cement (0.96 vol% of composite)	In this study
Carbon black		7.22–11.4 vol% of composite	[25]
Carbon fibers	3 mm—length 7.2 µm—diameter	0.28 vol% of composite	[6]
	12 mm—length 7.2 µm—diameter	0.09 vol% of composite	[6]
	5 mm—length 7.0 µm—diameter	1.2 wt% of cement	[9]
Carbon nanotubes		1 wt% of cement	[13]
		0.6 wt% of cement	[34]
Expanded graphite		5 wt% of cement	[20]
Graphene nanoplatelets		2.4 vol% of composite	[15]
		2 vol% of composite	[19]
		1.2 wt% of cement	[16]
Shungite		16.2 vol% of composite	[31]

**Table 3:** Comparison of sensitivity of cement composites with conductive additives.

	Conductive Additive	Content	GF	Stress Sensitivity, %/MPa	Reference
Cement composites	Exfoliated graphite	2 wt% of cement (0.96 vol% of composite)	77	0.38	In this study
	Carbon black	7 wt% of cement	30	-	[26]
	Carbon fibers	0.28 vol% of composite	31	-	[6]
	Carbon nanofibers	2 wt% of cement	50	-	[7]
	Carbon nanotubes	1.1 vol% of cement	220	-	[14]
	Carbon nanotubes and carbon fibers	0.6 wt% of cement	64	0.54	[58]
	Copper powder	0.35 vol% of composite	44	-	[33]
	Expanded graphite	5 wt% of cement	68	-	[21]
	Nanographite	5 vol% of cement	156	0.78	[19]
	Shungite	31 vol% of composite	29	0.38	[31]
	Steel fibers	0.8 vol% of composite	127	-	[28]
Metal foil strain gauge		-	2–5	-	-

**Table 4:** Seebeck coefficients of cement composites with different conductive additives.

	Conductive Additive	Content	Seebeck Coefficient, μV/K	Reference
Cement composites	Exfoliated graphite	3 wt% of cement (1.43 vol% of composite)	6.9	In this study
	Carbon fibers	0.97 vol% of composite (w/c = 0.44)	19.7	[4]
		3 wt% of cement (w/c = 0.5)	8.5	[8]
		1 wt% of cement (w/c = 0.3)	17.8	[9]
		1.2 wt% of cement (w/c = 0.3)	5.5	[9]
	Carbon nanotubes	15 wt% of cement	57.9	[11]
	Expanded graphite obtained at 500 °C	6 wt% of cement (w/c = 0.5; s/c = 0.75)	13.7	[22]
	Expanded graphite obtained at 1000 °C	4 wt% of cement (w/c = 0.5; s/c = 0.75)	3.0	[22]
	Graphene nanoplates	15 wt% of cement (w/c = 0.1; compressed samples)	34	[17]
	Graphite powder	30 wt% of cement (w/c = 0.5; s/c = 0.75)	18	[24]
	Reduced graphene oxide	5 vol% of cement (w/c = 0.2; compressed samples)	32.7	[18]
	Shungite	20 vol% of composite (w/c = 0.76; c/s = 0.75)	4.1	[31]
	Steel fibers	0.2 vol% of composite (w/c = 0.35)	59	[29]

## Data Availability

The data is contained within the article. Additional data are available on request from the corresponding author.
